# Suppressing the KIF20A/NUAK1/Nrf2/GPX4 signaling pathway induces ferroptosis and enhances the sensitivity of colorectal cancer to oxaliplatin

**DOI:** 10.18632/aging.202774

**Published:** 2021-03-26

**Authors:** Changshun Yang, Yu Zhang, Shengtao Lin, Yi Liu, Weihua Li

**Affiliations:** 1 Department of Surgical Oncology, Fujian Provincial Hospital, Fuzhou 350001, China; 2 Department of Pathology, The First Affiliated Hospital of Fujian Medical University, Fuzhou 350001, China; 3 Department of Endoscopy, National Cancer Center/Cancer Hospital, Chinese Academy of Medical Science and Peking Union Medical College, Beijing 100000, China

**Keywords:** colorectal cancer, KIF20A, oxaliplatin

## Abstract

Oxaliplatin resistance can develop in colorectal cancer (CRC), which may involve inhibition of ferroptosis, although further research is needed to understand this potential mechanism. We evaluated CRC cells with acquired oxaliplatin resistance (HCT116-Or) or congenital resistance (H716) to determine whether a ferroptosis inducer (RSL3) or inhibitor (liproxstatin-1) could modulate the effects of oxaliplatin. The results suggested that induction of ferroptosis could significantly reverse the oxaliplatin resistance of the CRC cells. Bioinformatic and cytobiological searches also revealed that KIF20A was highly expressed in the oxaliplatin-resistant cell lines and was strongly correlated with survival among CRC patients. Silencing KIF20A enhanced cellular sensitivity to oxaliplatin both *in vivo* and *in vitro*, and silencing KIF20A also suppressed NUAK1 activation, while a NUAK1 agonist (ETC-1002) could reverse the oxaliplatin sensitivity of KIF20A-silenced cells. Moreover, silencing NUAK1 up-regulated the expression of PP1β, down-regulated the phosphorylation of downstream GSK3βSer9, suppressed the nuclear import of Nrf2, inhibited the expression of a ferroptosis key negative regulatory protein (GPX4), and blocked cellular resistance. Applying a Nrf2 agonist (oltipraz) also reversed the oxaliplatin sensitivity of NUAK1-silenced cells. Therefore, cellular ferroptosis may be inhibited via the KIF20A/NUAK1/PP1β/GPX4 pathway in CRC cells, which may underly the resistance of CRC to oxaliplatin.

## INTRODUCTION

It is recommended by the National comprehensive cancer network (NCCN) guideline that, patients with middle and advanced stage CRC should be treated by the comprehensive therapeutic scheme dominated by surgical resection and supplemented by chemotherapy; besides, the oxaliplatin-based FOLFXOX and CapeOX schemes should be determined as the first-line chemotherapeutic scheme [1, 2]. Oxaliplatin is verified to extend the median disease-free survival (DFS) and overall survival (OS) of advanced CRC patients, but clinical data suggest that only less than 40% advanced CRC patients can benefit from it [3, 4]. This may be ascribed to the occurrence of resistance to oxaliplatin-based chemotherapeutic scheme, which can not further control cancer deterioration and progression. It has become the greatest obstacle that affects the effect of oxaliplatin on persistently promoting the survival time of advanced patients.

At present, with the deepening understanding of CRC biological and pathological basis, domestic and foreign basic studies regarding Oxaliplatin resistance have dug out a series of molecular mechanisms related to such phenomenon; for instance, activation of the ABC transporters and supermethylation of the CpG island [5–7]. On this basis, researchers have carried out therapeutic tests using a series of drugs combined with Oxaliplatin in basic and clinic, but no satisfactory results can be achieved, and long-term drug combination will markedly enhance the toxic and side reactions.

Ferroptosis is a brand new iron-dependent non-apoptotic way of cell death characterized by intracellular reactive oxygen species (ROS) accumulation, which has gradually become a research hotspot in the field of tumor resistance reversal [8, 9]. Such a death manner is resulted from the imbalanced intracellular lipid redox induced by small molecule drugs (generally including chemotherapeutics and targeted preparations), and its mechanism is markedly correlated with the inactivation or down-regulation of glutathione peroxidase (especially for GPX4) [10]. On the contrary, the up-regulation or activation of intracellular GPX4 level can induce cell resistance to ferroptosis, suppress the therapeutic effects of drugs, and finally result in tumor resistance to chemotherapeutics [11]. In multiple cancers (such as liver cancer, pancreatic cancer, and head and neck cancer), induced ferroptosis has been verified to reverse the resistance of numerous chemotherapeutics and targeted preparations (including Cisplatin and Sorafenib), enhance drug effects, and apparently prolong the survival of tumor-bearing mice [12–14]. Similar to other tumors, an increasing number of studies on the correlation of CRC with ferroptosis and key regulatory protein expression levels are reported in the recent two years. Research indicates that, the GPX4 levels in tumor tissues of advanced CRC patients are remarkably higher than those in para-carcinoma tissues [15]. Moreover, some laboratory reports that, a large dose of RSL3 (a ferroptosis inducer) can suppress GPX4 down-regulation, increase the production of intracellular lipid peroxide, and induce the death of CRC cells [16]. On this basis, our research group believed that the high GPX4 expression-mediated ferroptosis resistance might be the pathological foundation of CRC resistance to Oxaliplatin. However, the correlation of the two should be verified, while the potential molecular mechanism remains to be further explored. These are the research objectives of this article, and the research results can provide a new thinking for reversing CRC resistance in clinic, which can offer the new molecular theoretical foundation for carrying out the novel combined medication mode for antitumor in clinic.

## RESULTS

### Inducing ferroptosis enhanced the sensitivity of CRC to oxaliplatin

RSL3 is a well-known ferroptosis inducer, which can activate the ferroptosis process in multiple cancer cells through irreversibly inducing the inactivation of ferroptosis key regulatory protein GPX4 [16, 17]. In this part of results, we first selected RSL3 in combination with Oxaliplatin to observe the response of resistant cell line to chemotherapeutic. It could be observed from Figure 1A, 1B that, the xenografts constructed by the acquired resistant cell line HCT116-Or and congenital resistant cell line H716 had poor sensitivity to Oxaliplatin *in vivo*, while the combined application of ferroptosis inducer RSL3 enhanced the suppression of Oxaliplatin on xenograft growth. On this basis, ferroptosis suppressor liproxstatin-1 [18] could reverse the effect of RSL3. Further, we also discovered in experiment *in vitro* (Figure 1C, 1D) that, RSL3 had little effect on the cell viabilities of HCT116-Or and H716 at 0.5 μM. In addition, the sensitivities of HCT116-Or and H716 to Oxaliplatin could be induced by the application of RSL3, while the combined application of liproxstatin-1 could suppress such effect. Moreover, flow cytometry and LDH release assay results suggested that, Oxaliplatin (10μM) alone had low killing effect on resistant cells, but its combined application with RSL3 markedly induced cell death, while the addition of liproxstatin-1 could reverse such effect (Figure 1E, 1F and Supplementary Figure 1A, 1B).

**Figure 1:**
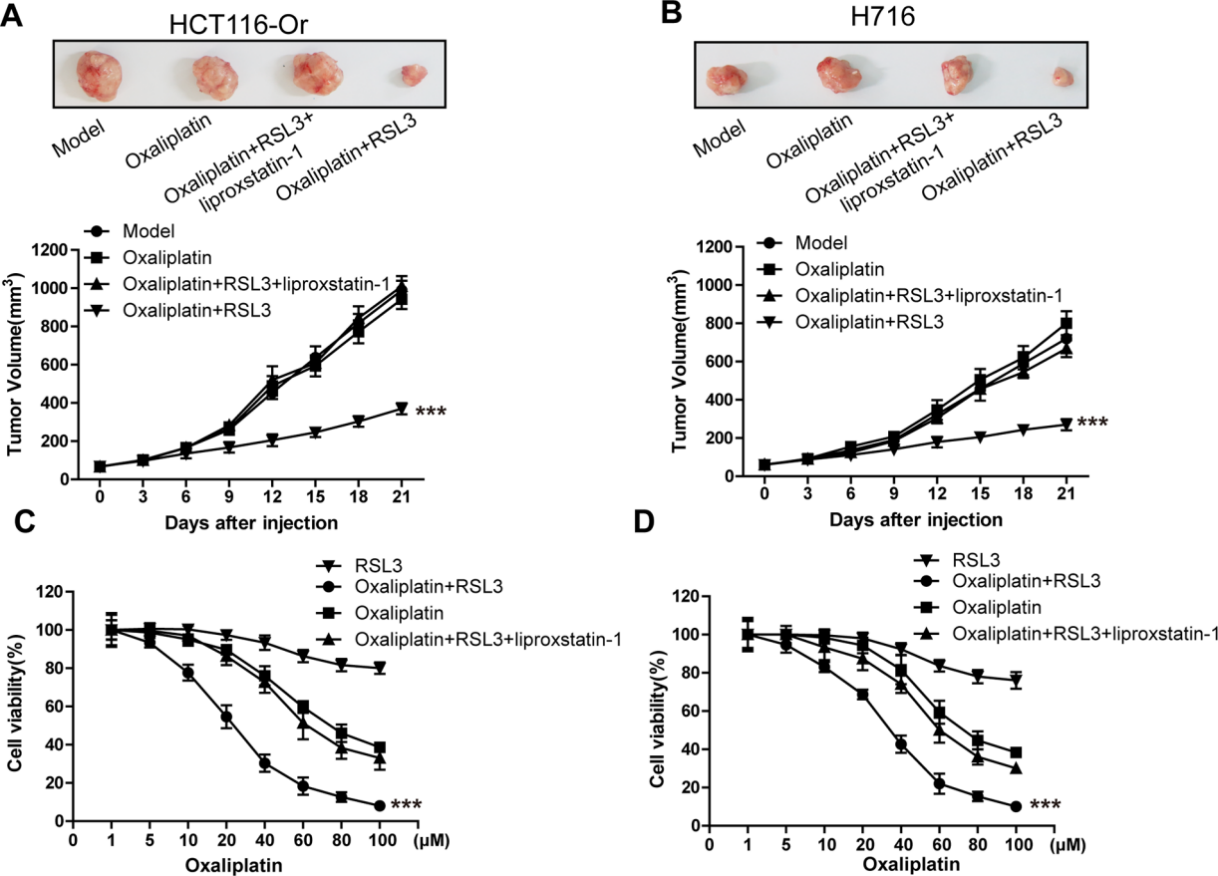
**Inducing ferroptosis enhanced the sensitivity of CRC to Oxaliplatin.** (**A**, **B**) HCT116-Or (**A**) and H716 (**B**) cells were selected to construct the subcutaneous xenograft model of nude mice, so as to observe whether RSL3 with or without liproxstatin-1 would affect the suppression of oxaliplatin on colorectal cancer *in vivo*. Top, representative images of xenografted tumor in the indicated groups. Bottom, statistical results of growth of xenografted tumor with time. The data are presented as the mean ± SD, ***p < 0.001 (versus Model). (**C**, **D**) The cell (HCT116-Or (**C**) and H716 (**D**)) viability was measured to observe whether RSL3 with or without liproxstatin-1 would affect the suppression of oxaliplatin on colorectal cancer *in vitro*. The data are presented as the mean ± SD, ***p < 0.001 (versus Oxaliplatin).

**Figure 1:**
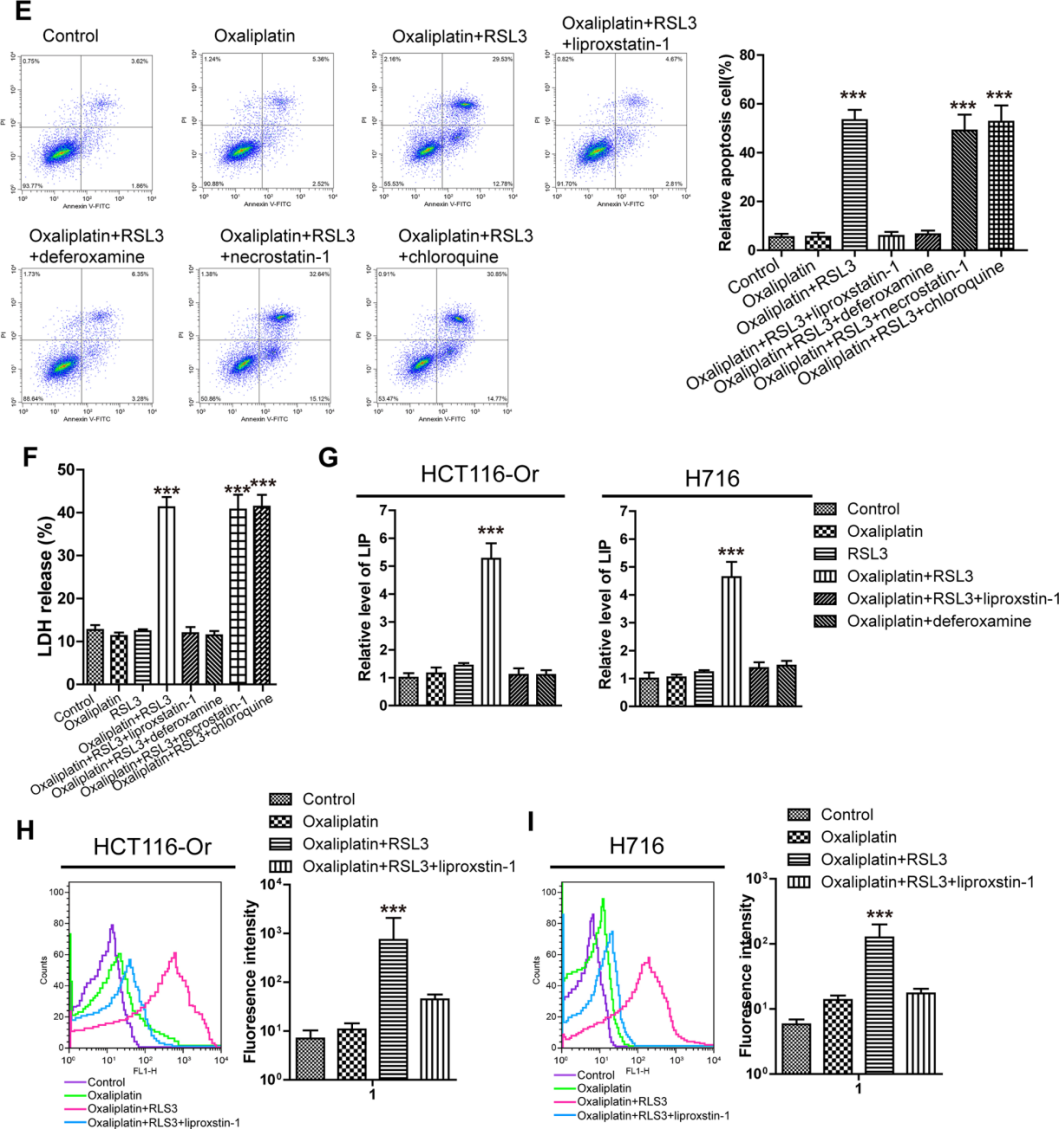
**Inducing ferroptosis enhanced the sensitivity of CRC to Oxaliplatin.** (**E**) Cell (HCT116-Or) death was assessed by flow cytometry (annexin V-FITC/PI staining) to observe whether RSL3 with or without the indicated inhibitors would affect the lethal effect of oxaliplatin on colorectal cancer *in vitro*. Left, representative results of annexin V-FITC/PI staining. Right, quantitative analysis. The data are presented as the mean ± SD, ***p < 0.001 (versus Oxaliplatin). (**F**) Cell (HCT116-Or) death was assessed by LDH release assay to observe whether RSL3 with or without the indicated inhibitors would affect the lethal effect of oxaliplatin on colorectal cancer *in vitro*. The data are presented as the mean ± SD, ***p < 0.001 (versus Oxaliplatin). (**G**) The cellular LIP was analyzed with a flow cytometer to observe whether RSL3 with or without liproxstatin-1 (or deferoxamine) would affect the LIP induction of oxaliplatin on colorectal cancer cells. Left, HCT116-Or cells. Right, H716 cells. The data are presented as the mean ± SD, ***p < 0.001 (versus oxaliplatin). (**H**, **I**) The cellular level of ROS (**H**) and lipid peroxidation (**I**) was assessed by flow cytometry to observe whether RSL3 with or without liproxstatin-1 would affect the oxidative damage induction of oxaliplatin on HCT116-Or cells. The data are presented as the mean ± SD, ***p < 0.001 (versus Oxaliplatin).

Subsequently, iron is the essential reaction element in numerous biological processes (including ferroptosis, together with ROS production and lipid peroxidation during this process), while LIP is the crossroad of cellular iron traffic, which has been reported to directly participate in the above biological processes [19, 20]. Therefore, the effects of Oxaliplatin alone and combined application with RSL3 (or liproxstatin-1) on the intracellular LIP level were detected. Our results (Figure 1G) suggested that Oxaliplatin alone had no obvious effect on LIP, while the combined application of RSL3 could evidently induce the intracellular LIP level, but such effect could be reversed by the further added liproxstatin-1 and deferoxamine. Afterwards, we also detected the effects of Oxaliplatin alone and combined application of RSL3 (or liproxstatin-1) on the ROS production and lipid peroxidation levels during the key biological processes of intracellular ferroptosis. Results of flow cytometry (Figure 1H, 1I and Supplementary Figure 1C, 1D) indicated that, Oxaliplatin alone had insignificant influence on inducing ROS and lipid peroxidation, while the combined application of RSL3 markedly induced the intracellular ROS and lipid peroxidation levels. However, such effect was reversed by the further added liproxstatin-1, and the trend was consistent with the LIP level.

Finally, to further verify the role of induced ferroptosis in enhancing the sensitivity of CRC to Oxaliplatin, we applied a series of cell death pathway inhibitors in combination with Oxaliplatin and RSL3. Our results (Figure 1E, 1F and Supplementary Figure 1A, 1B) indicated that, the ferroptosis inducer RSL3-mediated enhanced sensitivity of resistant cell line *in vitro* to Oxaliplatin was only reversed by deferoxamine (a iron chelating agent, 100μM), but it was not affected by necrostatin-1 (necrotic apoptosis inhibitor, 10μM) and chloroquine (autophagy inhibitor, 25μM).

### High KIF20A expression in resistant CRC cell line suppressed the intracellular ferroptosis process

To further explore the mechanism of ferroptosis-related CRC resistance, we first mined genes significantly related to GPX4, the key end effector of negative ferroptosis regulation, from the CRC patient samples in TCGA database using bioinformatic means (Figure 2A). Besides, correlation of the expression levels of these genes with patient prognosis and classification was further analyzed. Finally, KIF20A was identified, which was markedly up-regulated in the CRC samples in the database from stage I to stage III (Figure 2B). Then, we applied the WB approach to compare the protein expression between resistant and non-resistant CRC cell lines. The results (Figure 2C) indicated that, compared with the non-resistant CRC cell line, KIF20A expression in HCT116-Or and H716 cells was evidently increased. This revealed that KIF20A might take part in GPX4 expression in resistant cell line to suppress the intracellular ferroptosis process, thus inducing cell resistance to Oxaliplatin.

**Figure 2:**
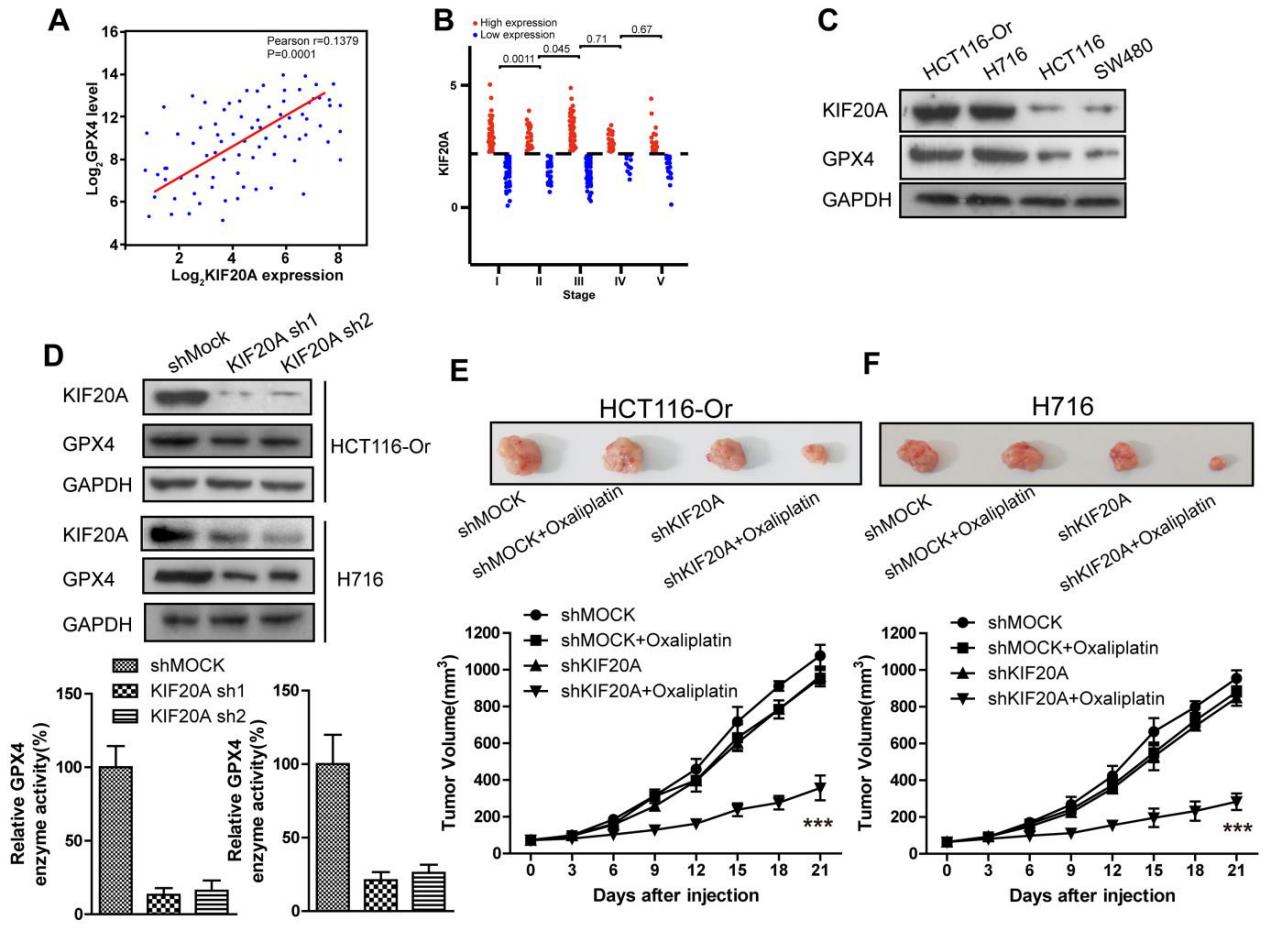
**High KIF20A expression in resistant CRC cell line suppressed the intracellular ferroptosis process.** (**A**) Correlation between expression levels of KIF20A and GPX4 in colorectal cancer samples. (**B**) The expression level of KIF20A of colorectal cancer patients in different stages. (**C**) The expression level of KIF20A in different colorectal cancer cell lines were examined by WB assay. (**D**) WB assay was used to observe whether KIF20A silencing could impact the intracellular GPX4 expression level. Top, HCT116-Or cells. Bottom, H716 cells. (**E**, **F**) HCT116-Or (**E**) and H716 (**F**) cells were selected to construct the subcutaneous xenograft model of nude mice, so as to observe whether KIF20A silencing would affect the suppression of oxaliplatin on colorectal cancer *in vivo*. Top, representative images of xenografted tumor in the indicated groups. Bottom, statistical results of growth of xenografted tumor with time. The data are presented as the mean ± SD, ***p < 0.001 (versus shMOCK+Oxaliplatin).

To verify the above-mentioned hypothesis, shRNA was selected to silence KIF20A in two resistant cell lines. The results (Figure 2D) suggested that, KIF20A silencing suppressed the intracellular expression level and activity of GPX4. *In vivo* xenograft experiment (Figure 2E, 2F) indicated that, compared with shMOCK cell line, Oxaliplatin evidently suppressed the growth of mouse xenograft formed by the shKIF20A cell line. Besides, *in vitro* cell viability (Figure 2G, 2H) results demonstrated that, KIF20A silencing could enhance the inhibitory effect of Oxaliplatin on HCT116-Or and H716, which could be reversed by the addition of liproxstatin-1 and deferoxamine. In addition, death detection (Figure 2I, 2J and Supplementary Figure 2A, 2B) results demonstrated that, KIF20A silencing reversed the resistance of CT116-Or and H716 to Oxaliplatin. Flow cytometry results revealed that, compared with shMOCK cell line, Oxaliplatin notably induced ROS production (Figure 2L and Supplementary Figure 2D), lipid peroxidation (Figure 2M and Supplementary Figure 2E) and LIP level (Figure 2K and Supplementary Figure 2C) in shKIF20A cell line. Further, the above experimental results were reversed by the addition of liproxstatin-1.

**Figure 2:**
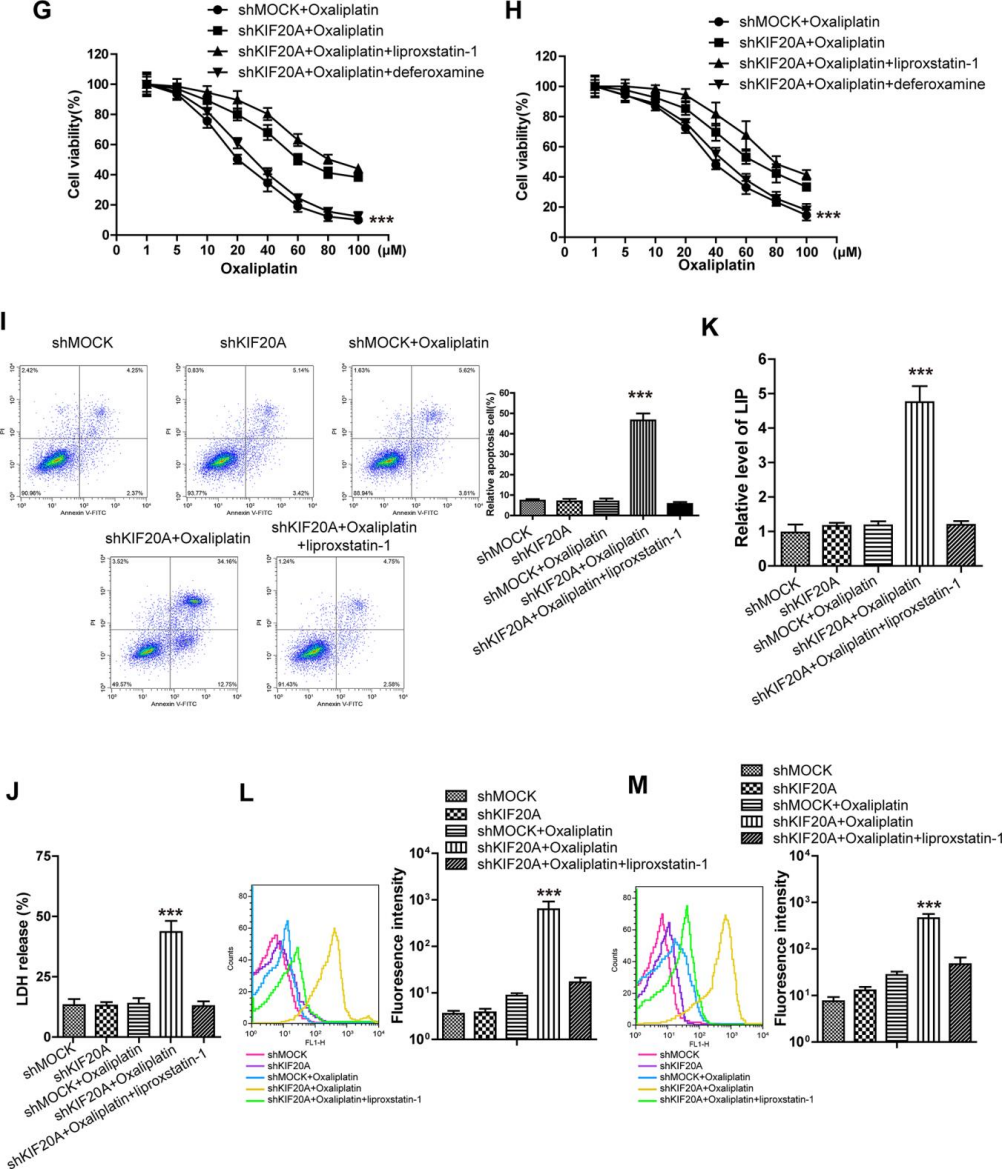
**High KIF20A expression in resistant CRC cell line suppressed the intracellular ferroptosis process.** (**G**, **H**) The cell (HCT116-Or (**G**) and H716 (**H**)) viability was measured to observe whether KIF20A silencing with or without liproxstatin-1 would affect the suppression of oxaliplatin on colorectal cancer *in vitro*. The data are presented as the mean ± SD, ***p < 0.001 (versus shMOCK+Oxaliplatin). (**I**) Cell (HCT116-Or) death was assessed by flow cytometry (annexin V-FITC/PI staining) to observe whether KIF20A silencing with or without liproxstatin-1 would affect the lethal effect of oxaliplatin on colorectal cancer *in vitro*. Left, representative results of annexin V-FITC/PI staining. Right, quantitative analysis. The data are presented as the mean ± SD, ***p < 0.001 (versus shMOCK+Oxaliplatin). (**J**) Cell (HCT116-Or) death was assessed by LDH release assay to observe whether KIF20A silencing with or without liproxstatin-1 would affect the lethal effect of oxaliplatin on colorectal cancer *in vitro*. The data are presented as the mean ± SD, ***p < 0.001 (versus shMOCK+Oxaliplatin). (**K**) The cellular LIP was analyzed with a flow cytometer to observe whether KIF20A silencing with or without liproxstatin-1 would affect the LIP induction of oxaliplatin on HCT116-Or cells. The data are presented as the mean ± SD, ***p < 0.001 (versus shMOCK+Oxaliplatin). (**L**, **M**) The cellular level of ROS (**L**) and lipid peroxidation (**M**) was assessed by flow cytometry to observe whether KIF20A silencing with or without liproxstatin-1 would affect the oxidative damage induction of oxaliplatin on HCT116-Or cells. The data are presented as the mean ± SD, ***p < 0.001 (versus shMOCK+Oxaliplatin).

### KIF20A induced NUAK1 activation to up-regulate GPX4 level, thus inducing CRC resistance to oxaliplatin

To further explore the molecular mechanism of KIF20A in regulating GPX4 level and resistance, the String database [21] was utilized to mine genes and proteins significantly interacted with KIF20A. The results (Figure 3A) suggested that, NUAK1, a kinase already verified to be related to the malignant progression and poor prognosis of CRC cells and the maintenance of intracellular redox balance [22], might potentially interact with KIF20A. To prove this result, shRNA was used to silence KIF20A in two resistant cell lines. WB results (Figure 3B) indicated that, KIF20A silencing inhibited the activation of intracellular NUAK1 (expressed as the phosphorylation level of MYPT1^S445^), but there was no effect on NUAK1 expression level [23]. Besides, we had utilized the pharmacological approaches (NUAK1 activator ETC-1002) to activate that kinase. Cell viability and death detection results (Figure 3C–3F) suggested that, ETC-1002 reversed the KIF20A silencing-induced enhanced sensitivity of resistant cell lines to Oxaliplatin. The subsequent flow cytometry results demonstrated that, ETC-1002 reversed Oxaliplatin-induced up-regulation of intracellular ROS (Figure 3H), lipid peroxidation (Figure 3I) and LIP level (Figure 3G) in the KIF20A silencing resistant cell lines.

**Figure 3:**
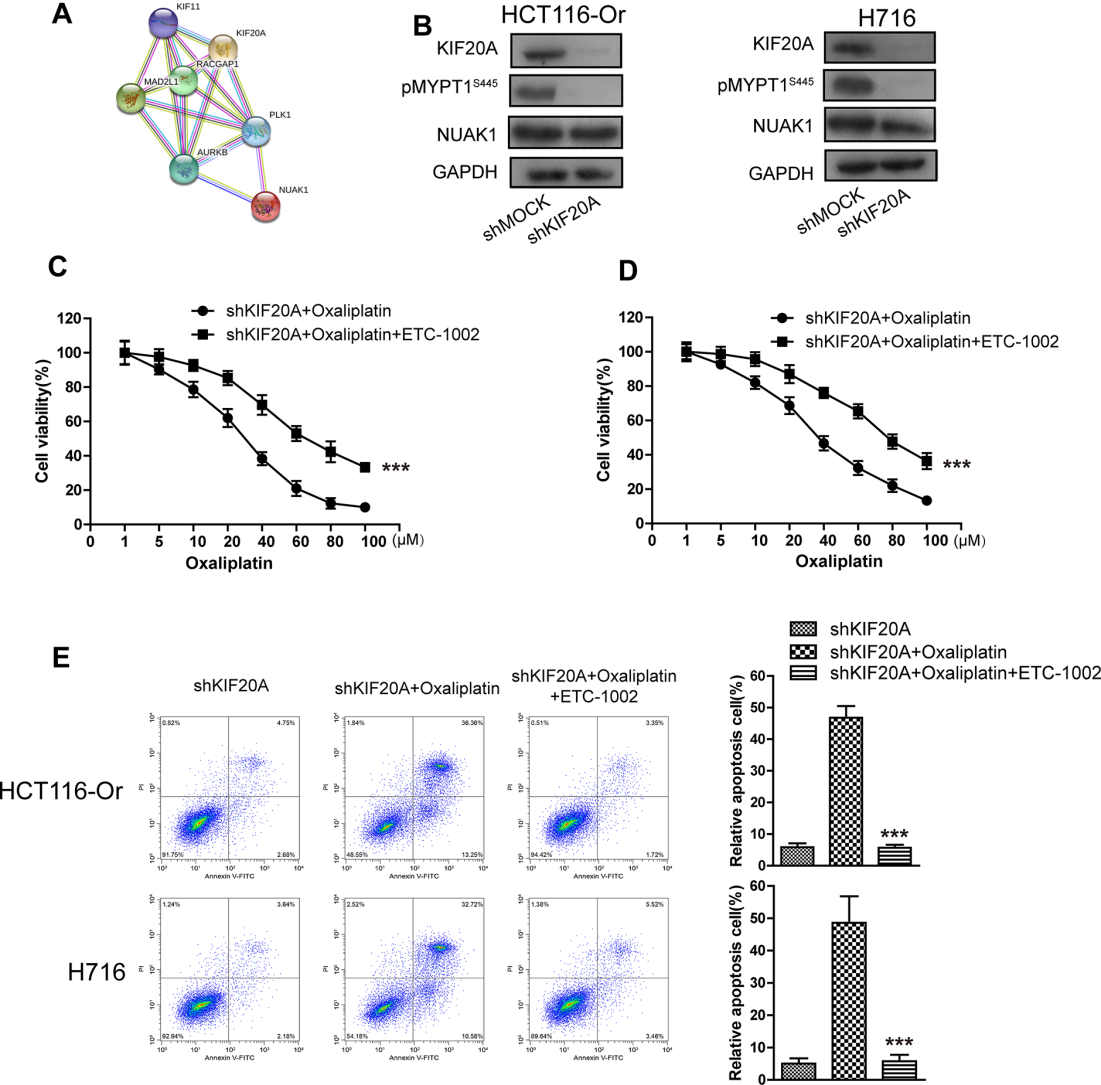
**KIF20A induced NUAK1 activation to up-regulate GPX4 level, thus inducing CRC resistance to Oxaliplatin.** (**A**) The protein-protein interaction between KIF20A and NUAK1 was screened out by String database. (**B**) WB assay was used to observe whether KIF20A silencing could impact the phosphorylation level of MYPT1^S445^. Left, HCT116-Or cells. Right, H716 cells. (**C**, **D**) The cell (HCT116-Or (**C**) and H716 (**D**)) viability was measured to observe whether ETC-1002 would affect the suppression of oxaliplatin on KIF20A-silenced colorectal cancer cells *in vitro*. The data are presented as the mean ± SD, ***p < 0.001 (versus shKIF20A+Oxaliplatin). (**E**) Cell death was assessed by flow cytometry (annexin V-FITC/PI staining) to observe whether ETC-1002 would affect the lethal effect of oxaliplatin on KIF20A-silenced colorectal cancer cells *in vitro*. Left, representative results of annexin V-FITC/PI staining. Right, quantitative analysis. Top, HCT116-Or cells. Bottom, H716 cells. The data are presented as the mean ± SD, ***p < 0.001 (versus shKIF20A+Oxaliplatin).

**Figure 3:**
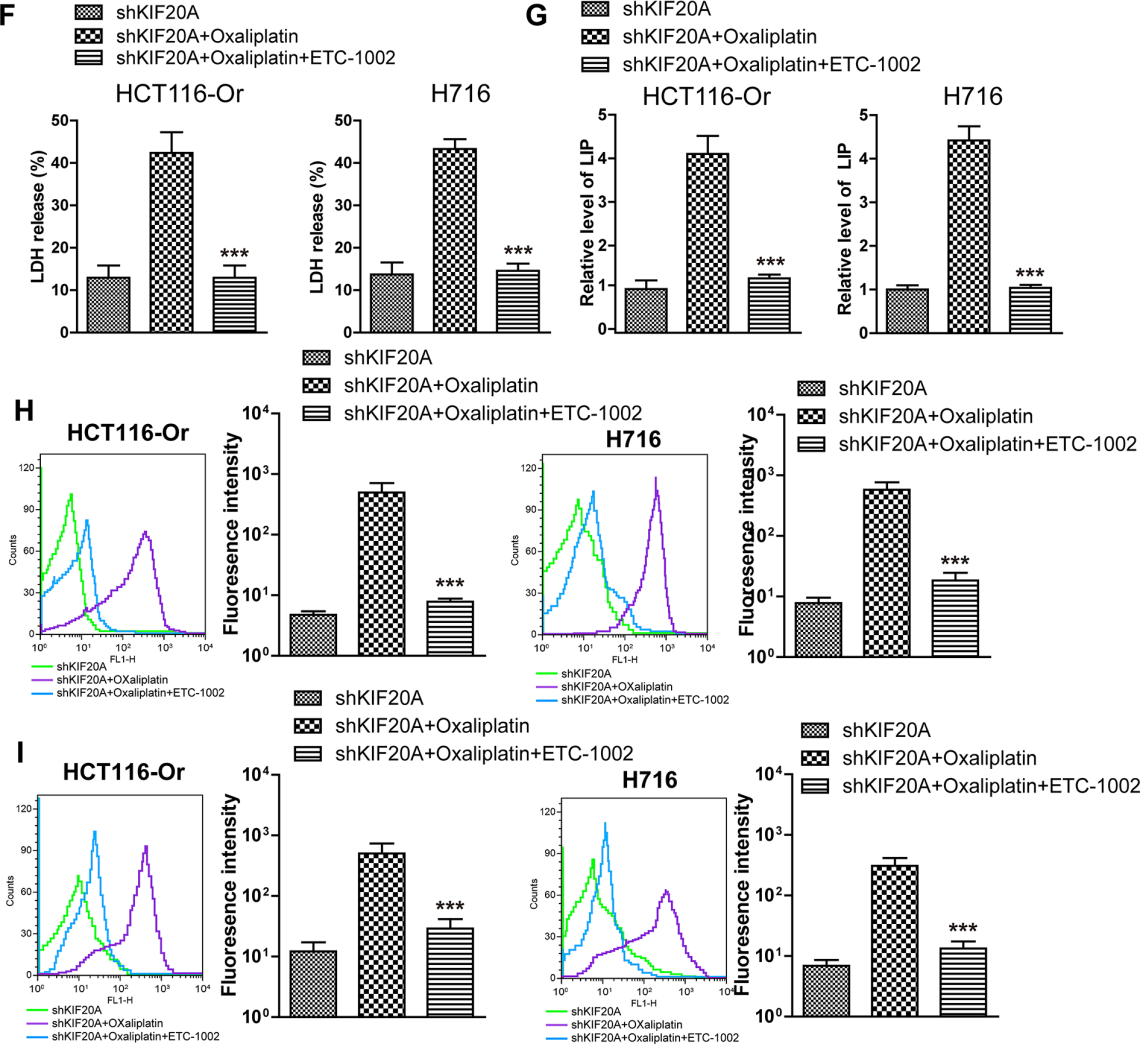
**KIF20A induced NUAK1 activation to up-regulate GPX4 level, thus inducing CRC resistance to Oxaliplatin.** (**F**) Cell death was assessed by LDH release assay to observe whether ETC-1002 would affect the lethal effect of oxaliplatin on KIF20A-silenced colorectal cancer cells *in vitro*. Top, HCT116-Or cells. Bottom, H716 cells. The data are presented as the mean ± SD, ***p < 0.001 (versus shKIF20A+Oxaliplatin). (**G**) The cellular LIP was analyzed with a flow cytometer to observe whether ETC-1002 would affect the LIP induction of oxaliplatin on KIF20A-silenced colorectal cancer cells. Left, HCT116-Or cells. Right, H716 cells. The data are presented as the mean ± SD, ***p < 0.001 (versus shMOCK+Oxaliplatin). (**H**, **I**) The cellular level of ROS (**H**) and lipid peroxidation (**I**) was assessed by flow cytometry to observe whether ETC-1002 would affect the oxidative damage induction of oxaliplatin on KIF20A-silenced colorectal cancer cells. Left, HCT116-Or cells. Right, H716 cells. The data are presented as the mean ± SD, ***p < 0.001 (versus shKIF20A+Oxaliplatin).

### The GSK3β/Nrf2 pathway mediated KIF20A/NUAK1 activation to induce the resistance of CRC resistant cell lines to oxaliplatin

To further explore the molecular mechanism of KIF20A/NUAK1 activation-induced CRC cell resistance, related literature was reviewed, and it was discovered that, NUAK1 inactivation in multiple cancer cells suppressed the H_2_O_2_-induced Nrf2 nuclear import, accelerated the imbalance of intracellular redox, and led to tumor cell death [24, 25]. In addition, the sensitivities of HCT116-Or and H716 to Oxaliplatin could be induced by the application of ML385 (Nrf2 inhibitor) (Supplementary Figure 3). As a result, we speculated that KIF20A-induced excessive activation of NUAK1 might induce the nuclear import and transcription of Nrf2 in resistant cell lines, maintain the intracellular redox balance, and suppress ferroptosis, thus inducing resistance. To verify such speculation, we first applied HTH-01-015 [26] (10μM) to inactivate NUAK1 in two resistant cell lines, and used WB to detect the Oxaliplatin-induced intranuclear Nrf2 level. The results (Figure 4A, 4B) suggested that, the small molecular inhibitor of NUAK1 suppressed Oxaliplatin-induced Nrf2 nuclear translocation. Moreover, PCR assay was also carried out to detect the effect of NUAK1 inactivation on the Oxaliplatin-induced Nrf2 transcription activity (Figure 4C, 4D). The results suggested that, this small molecular inhibitor can inhibit the mRNA levels of Nrf2 downstream signal molecules, such as GCLC and GCLM in resistant cell lines. In addition, HTH-01-015 could also inhibit the mRNA level of GPX4. Besides, the methylation of Nrf2 cytoplasm suppressor protein Keap1 was induced through pharmacological approach (Nrf2 agonist 4-Octyl Itaconate [27]), so as to induce Nrf2 expression and nuclear import. Cell viability and death detection results (Figure 4E, 4H) demonstrated that, 4-Octyl Itaconate (60 μM) reversed NUAK1 silencing-induced enhanced sensitivity of resistant cell lines to Oxaliplatin. The subsequent results revealed that, 4-Octyl Itaconate could suppress Oxaliplatin-induced up-regulation of ROS (Figure 4K), lipid peroxidation (Figure 4L) and LIP levels (Figure 4I, 4J) in NUAK1 silencing resistant cell lines.

**Figure 4:**
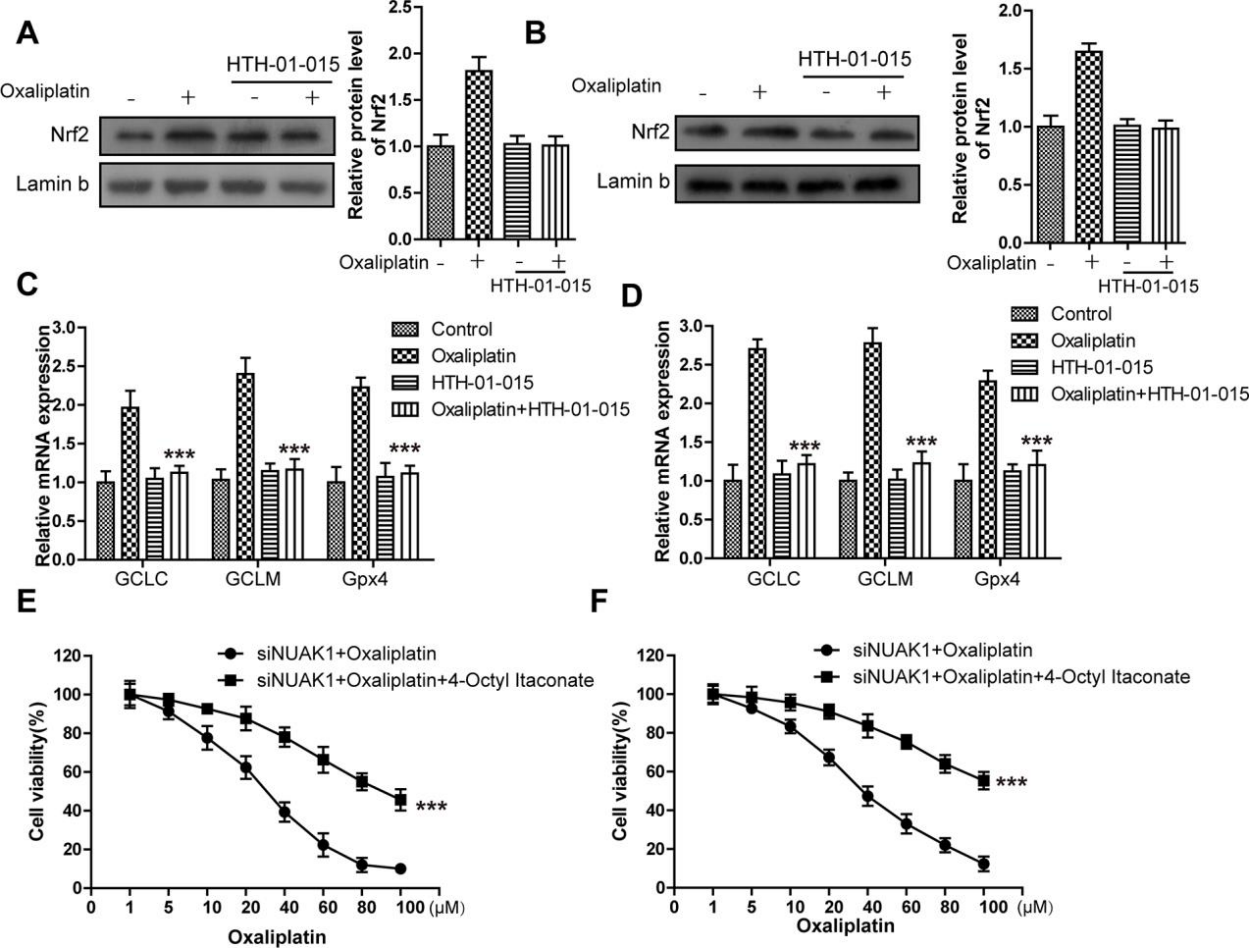
**KIF20A/NUAK1 induce the resistance of CRC resistant cell lines to Oxaliplatin through activating Nrf2 pathway.** (**A**, **B**) Immunoblots of Nrf2 protein levels in nuclear extracts from HCT116-Or (**A**) and H716 (**B**) cells after treatment with oxaliplatin, with and without prior depletion of NUAK1 by HTH-01-015 (10 μmol/L). (**C**, **D**) The mRNA levels of GCLC, GCLM and GPX4 were examined by PCR assay to observe whether NUAK1 depletion could affect the intracellular transcriptional activity of Nrf2 in HCT116-Or (**C**) and H716 (**D**) cells. The data are presented as the mean ± SD, ***p < 0.001 (versus Oxaliplatin). (**E**, **F**) The cell (HCT116-Or (**E**) and H716 (**F**)) viability was measured to observe whether 4-Octyl Itaconate would affect the suppression of oxaliplatin on NUAK1-silenced colorectal cancer cells *in vitro*. The data are presented as the mean ± SD, ***p < 0.001 (versus siNUAK1+Oxaliplatin).

**Figure 4:**
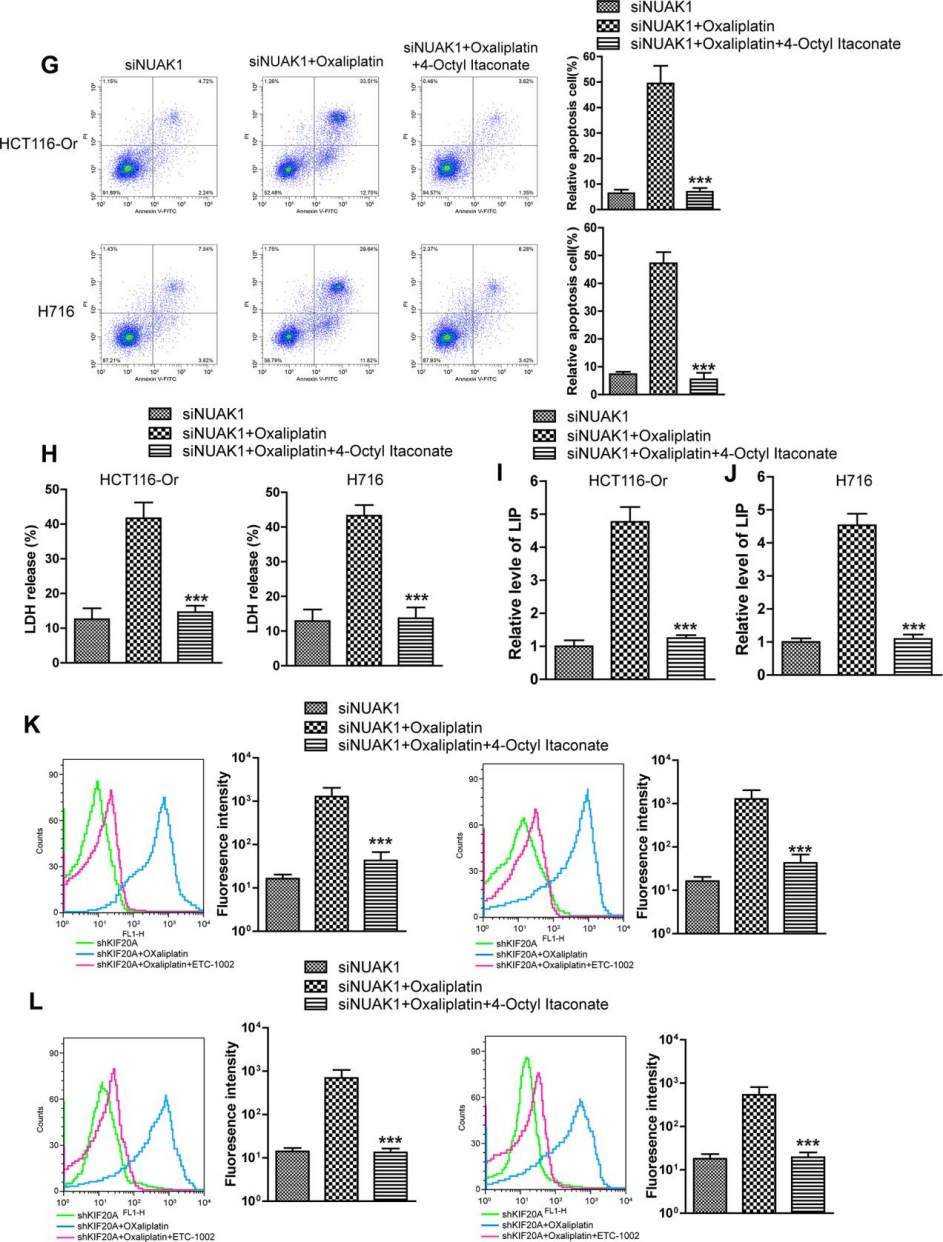
**KIF20A/NUAK1 induce the resistance of CRC resistant cell lines to Oxaliplatin through activating Nrf2 pathway.** (**G**) Cell death was assessed by flow cytometry (annexin V-FITC/PI staining) to observe whether 4-Octyl Itaconate would affect the lethal effect of oxaliplatin on NUAK1-silenced colorectal cancer cells *in vitro*. Left, representative results of annexin V-FITC/PI staining. Right, quantitative analysis. Top, HCT116-Or cells. Bottom, H716 cells. The data are presented as the mean ± SD, ***p < 0.001 (versus siNUAK1+Oxaliplatin). (**H**) Cell death was assessed by LDH release assay to observe whether 4-Octyl Itaconate would affect the lethal effect of oxaliplatin on NUAK1-silenced colorectal cancer cells *in vitro*. Top, HCT116-Or cells. Bottom, H716 cells. The data are presented as the mean ± SD, ***p < 0.001 (versus siNUAK1+Oxaliplatin). (**I**, **J**) The cellular LIP was analyzed with a flow cytomete to observe whether 4-Octyl Itaconate would affect the LIP induction of oxaliplatin on NUAK1-silenced colorectal cancer cells. (**I**) HCT116-Or cells. (**J**) H716 cells. The data are presented as the mean ± SD, ***p < 0.001 (versus siNUAK1+Oxaliplatin). (**K**, **L**) The cellular level of ROS (**K**) and lipid peroxidation (**L**) was assessed by flow cytometry to observe whether 4-Octyl Itaconate would affect the oxidative damage induction of oxaliplatin on NUAK1-silenced colorectal cancer cells. Left, HCT116-Or cells. Right, H716 cells. The data are presented as the mean ± SD, ***p < 0.001 (versus siNUAK1+Oxaliplatin).

**Figure 5:**
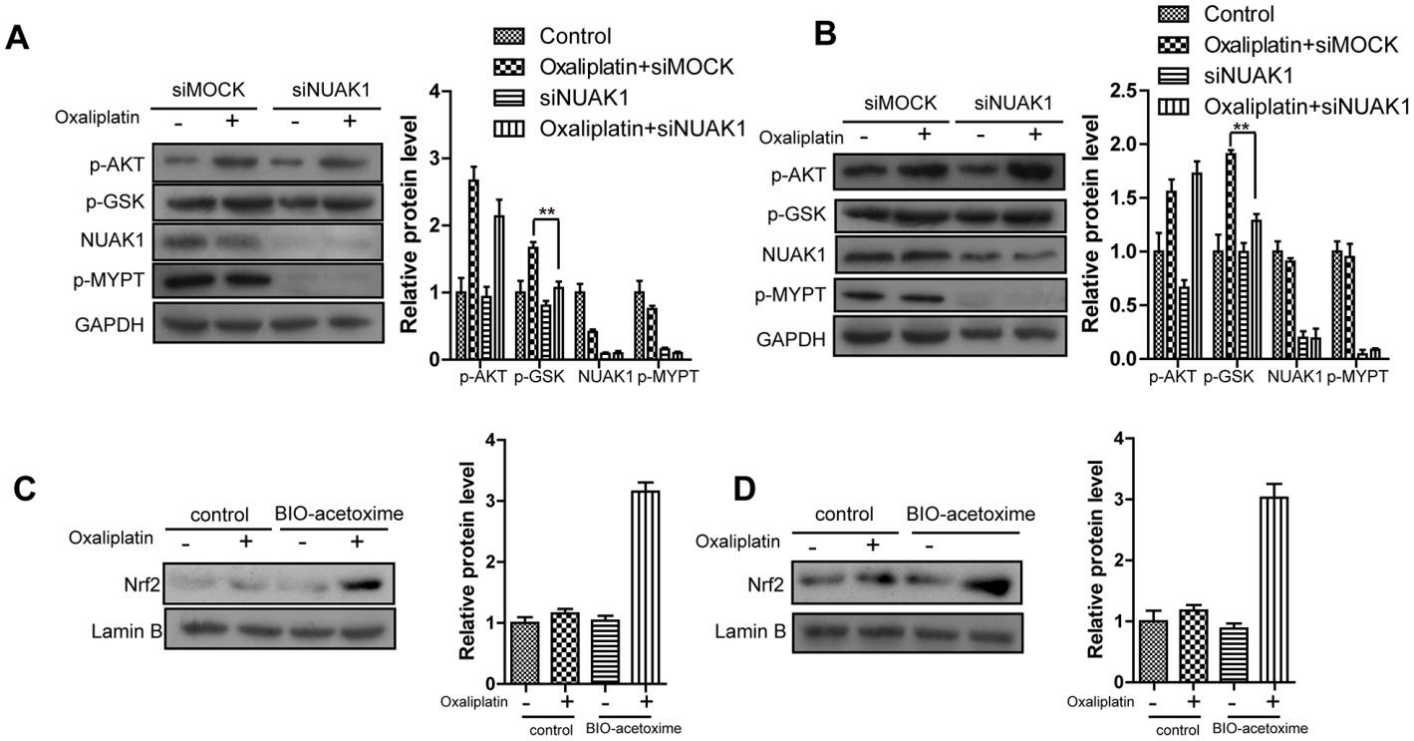
**NUAK1 promotes nuclear translocation of NRF2 in colorectal cancer cell by antagonizing GSK3a.** (**A**, **B**) Immunoblots of NUAK1-depleted or control colorectal cancer cell ((HCT116-Or (**A**) and H716 (**B**)) cytosolic fractions after treatment with oxaliplatin. (**C**, **D**) Pretreatment of NUAK1-depleted colorectal cancer cells ((HCT116-Or (**C**) and H716 (**D**)) with GSK3β inhibitor BIO-acetoxime (1 μmol/L for 6 hours) restores oxaliplatin-induced NRF2 nuclear translocation.

As is well known, AKT activation-induced GSK3β^Ser9^ phosphorylation can promote the nuclear import of Nrf2 under the stimulation of oxidative stresses (including ROS and multiple chemotherapeutics) [28]. Consequently, we detected whether NUAK1 affected the AKT/GSK3β pathway to regulate the nuclear translocation of Nrf2. WB experimental results (Figure 5A, 5B) revealed that, Oxaliplatin markedly induced the phosphorylation of AKT and GSK3β^Ser9^ in HCT116-Or and H716 cells. However, NUAK1 silencing had no influence on the Oxaliplatin-induced AKT activation, but it could reverse GSK3β^Ser9^ phosphorylation. The above results revealed that, NUAK1 suppressed the de-phosphorylation pathway of GSK3β^Ser9^ in resistant cell lines to maintain the phosphorylation status of that site. By contrast, small molecular inhibitor BIO-acetoxime (GSK3β inhibitors) pretreatment (1 μmol/L for 6h) [29] reversed the down-regulated intranuclear expression of Nrf2 induced by NUAK1 silencing in the resistant cell lines (Figure 5C, 5D).

## DISCUSSION

At present, with the increasing understanding towards the CRC biological and pathological foundation, a series of molecular mechanisms related to Oxaliplatin resistance have been dug out in domestic and foreign studies; for instance, the excessive activation of DNA damage repair system, over-expression of the membrane transporter, autophagy resistance of CRC cells, activation of the metalloprotease family-dependent EGFR, LPCAT2-mediated intracellular lipid droplet accumulation, and super-methylation of CpG island [30–32]. On this basis, researchers have carried out basic and clinical treatment experiments using a series of drugs in combination with Oxaliplatin, but the results are not satisfactory, and long-term drug combination will markedly increase the toxic and side effects. For instance, Cetuximab, a EGFR-targeting inhibitor, was used in a large phase III clinical trial in Europe, and the experimental data suggested that, FOLFOX4+Cetuximab (n=791) could not extend the disease-free survival (DFS) of stage III CRC patients after surgical resection, which was true even for KRAS wild-type patients [33]. Similarly, no positive clinical result is obtained from Azacitidine (combined CAPOX regimen) that targets the DNA methylation mechanism [34]. Consequently, how to reverse the Oxaliplatin resistance, better exert its anti-CRC effect in a more durable manner, and extend patient survival, has aroused the great attention from clinicians and researchers.

With the extensive research on CRC pathophysiology, the CRC resistance mechanism is increasingly discussed. Ferroptosis is a brand new iron-dependent non-apoptotic way of cell death characterized by intracellular reactive oxygen species (ROS) accumulation, which has gradually become a research hotspot in the field of tumor resistance reversal. Inducing ferroptosis in multiple cancers has been verified to reverse the resistance of multiple chemotherapeutics and targeted preparations, and enhance the drug therapeutic effects. On this basis, we had proposed a hypothesis that ferroptosis resistance in CRC cells might mediate the resistance of cancer cells to Oxaliplatin. Therefore, in this study, we had first applied the *in vivo* and *in vitro* models to verify that, the application of ferroptosis inducer RSL3 could increase the sensitivity of resistance CRC cell lines to Oxaliplatin, suppress xenograft growth, kill tumor cells, up-regulate intracellular LIP level, induce ROS accumulation and lipid peroxidation. Moreover, such effect could be reversed by two ferroptosis inhibitors (liproxstatin-1 and deferoxamine).

Afterwards, the large sample database (TCGA) was utilized for bioinformatic mining, with an attempt to search for genes significantly correlated with GPX4, the key end effector of negative ferroptosis regulation. Besides, the correlation of the expression levels of these genes with the prognosis and classification of CRC patients was further analyzed. It could be seen from these results that, KIF20A was identified. KIF20A, a member of the kinesin (KIF) family located in chromosome 5q31, can encode the mitosis kinesin-like molecule, possesses the highly conserved dynamic domain, has ATP activity, and can move towards the microtubule anode. Such gene exerts its effect in meiosis telophase as a microtubule-related motor protein, in the meantime, it participates in mediating vesicle transport from Golgi complex to endoplasmic reticulum at interkinesis [35]. That gene has been verified to be closely correlated with the genesis and development of multiple tumors, participate in the pathological process of multiple tumors, and its high expression is markedly correlated with dismal prognosis [36–39].

TCGA database analysis results suggested that, that gene was apparently up-regulated in CRC samples from the database with the increasing malignant grade of patient classification. Besides, the median expression of that gene was used to divide the samples into high and low expression groups, and difference in OS between these two groups was significant. Subsequent WB experiment verified that, compared with the non-resistant CRC cell line, KIF20A expression was dramatically increased in resistant cell line. Moreover, the subsequent forward-backward verification experiment proved that, high KIF20A expression in resistant cell lines up-regulated the intracellular GPX4 expression to maintain the intracellular redox balance, suppress the ferroptosis process, and induce cell resistance to Oxaliplatin.

To further explore the mechanism of KIF20A-mediated CRC cell line resistance (ferroptosis resistance), this research group had applied bioinformatic and molecular biological means in combination and mined NUAK1, the potential downstream of KIF20A. The research results suggested that, KIF20A silencing suppressed NUAK1 activation, while stimulating the activation of that kinase through pharmacological means could reverse KIF20A silencing-induced enhanced sensitivity of resistant cell lines to Oxaliplatin. Afterwards, our research results verified that, the abnormal activation of NUAK1 in resistant CRC cells suppressed PP1β activity, and regulated the phosphorylation level of GSK3βS9 site to induce the nuclear import and transcription activity of Nrf2, up-regulate the levels of intracellular series anti-oxidative molecules, maintain the redox balance in cancer cells, and induce cancer cell resistance. The imbalance of cellular redox status was closely correlated with the activities of anti-cancer drugs (including multiple chemotherapeutics and targeted preparations), while the over-expression and high transcription activity of Nrf2 in tumor cells were verified to participate in the resistance process, leading to the poor patient prognosis.

It can be figured out based on previous exploration and existing basic research reports, the excessive expression and activation of KIF20A/NUAK1 in CRC cells can suppress the Oxaliplatin-induced intracellular redox imbalance and ferroptosis, and induce cell resistance to chemotherapeutics through the GSK3β/Nrf2 pathway. It shows high research value and prospect as a new target in resistance reversal treatment.

## MATERIALS AND METHODS

### Cell culture and drugs

HCT116 and H716 cells [7] were purchased from ATCC. Before use, these cell lines were identified through genetic and epigenetic labels, and detected routinely for mycoplasma pollution. Two cell lines were cultured in the DMEM supplemented with 10% fetal bovine serum (FBS) and 1% penicillin/streptomycin in the constant temperature incubator under 5% CO_2_ and 37° C. HCT116 cells were used to construct the homogene acquired Oxaliplatin-resistant cell line; in brief, HCT116 cells were exposed to the medium containing increasing Oxaliplatin concentrations, and finally HCT116-Or resistant to 10 μM Oxaliplatin was screened. These two cell lines were obtained from ATCC over the past 2-3 years, and periodically identified using a set of STR labels as well as a set of genes with known genetic and epigenetic features at an interval of 4-6 months. The final identification was carried out in November 2018. Oxaliplatin was purchased from Sigma.

### Nude mouse xenograft model

The 5-6-week-old female SCID nude mice were purchased from Model Animal Research Center of Nanjing University, raised in the SPF ventilating cages at the light/dark cycle of 12/12 h (light on 7:00 a.m. to 7:00 p.m.), and fed with ordinary diet or high-fat diet according to the model requirements. HCT116-Or or H716 cells (7*10^6^) were suspended into 200 μl matrigel (BD Bioscience), and injected into the subcutaneous tissues of mouse right lower limbs. The growth of subcutaneous xenograft was monitored using the vernier caliper at an interval of 2 weeks, and the tumor volume was calculated according to the formula=0.5×length ×width^2^. Animals were grouped randomly (eight mouse per group) and medication was initiated when the average xenograft size was over 100 mm3: Oxaliplatin was given alone weekly through intraperitoneal injection (5 mg/kg), or in combination with liproxstatin-1 through intraperitoneal injection for twice a week (125 mg/kg) and RSL3 through intra-tumor injection (100 mg/kg, in order to achieve better local concentration and reduce the probable systemic toxicity of RSL3) weekly. The animal and xenograft growth states were recorded every week, and the animals were regarded as dead when the xenograft volume was > 2000 mm^3^. Then, the animals were sacrificed through CO_2_ suffocation, and the xenografts were removed and preserved in liquid nitrogen for subsequent molecular biological experiments.

### Cell viability detection

To detect the sensitivity of CRC cells regulated by multiple small molecule inhibitors and agonists *in vitro* to Oxaliplatin, HCT116-Or and H716 cells (wild type or gene modification type) were planted into the 96-well plates (Corning) at the density of 6000/well, and the medium was removed 24 h after cell balance. Afterwards, cells were further cultured for 24 h with medium containing various concentrations of Oxaliplatin alone or in combination with various pathway regulators that did not affect tumor cell growth at appropriate concentrations (Oltipraz 40 μM, ETC-1002 30 μM, liproxstatin-1 1 μM, ML385 5 μM, and RSL3 0.5 μM). All drugs were prepared into appropriate concentrations using DMSO, and the medium in each well contained equivalent amount of DMSO. CellTiter-Glo reagent (Promega) was added into the cell medium in accordance with the manufacturer instruction, the absorbance value was read using the BioTek Synergy 96-well microplate reader, and all data were repeated for three times.

### Cell death determination

The cell death level was detected using the Pharmingen annexin V-FITC detection kit (BD, United States) according to the manufacturer instruction. Then, cells were counted and seeded into the 6-well plate at the density of 10^6^ cells/well. After standing for 12 h, cells were washed with PBS, and later medium containing 5 μM Oxaliplatin alone or various pathway regulators (Oltipraz 40 μM, ETC-1002 30 μM, liproxstatin-1 1 μM, and RSL3 0.5 μM) was added to culture for additional 24 h. After trypsin digestion, all cells in the wells were collected, including the suspending dead cells. Later, cells were centrifuged and washed repeatedly, followed by resuspension using the 4° C binding buffer until the concentration was 1×10^6^/ml. 100 μl cell suspension was collected and mixed with 5 μl FITC annexin V as well as 5 μl PI fluorochrome. The mixed solution was incubated for 15 min at room temperature in dark, then cells were analyzed and counted using the FACS Calibur flow cytometer.

### LDH release assay

HCT116-Or and H716 cells were treated as described above, then the supernatant were collected by centrifugation (2000g, 20 min), and the LDH release was evaluated using an LDH assay kit according to the protocol instruction. Thereafter, absorbance was detected at wavelength of 490 nm, and levels of LDH released were normalized to the control group.

### Detection of labile iron pool (LIP)

LIP was detected according to the method described in manufacturer instruction. In brief, cells were treated with drugs in the 6-well plates for 12 h, digested with trypsin, and washed with PBS. Later, cells were resuspended with PBS until the concentration of 1×10^6^/ml, and incubated with 0.05 μM calcein-acetoxymethyl ester (AnaSpec) at room temperature for 15 min. Subsequently, PBS was used to elute the dye, and cells were incubated with deferiprone (100 μM) at 37° C for 1 h or not subjected to incubation, followed by analysis using the flow cytometer. The fluorescence was measured at the wavelength of 525 nm, and the difference in average fluorescence between incubation with and without deferiprone was compared to reflect the intracellular LIP level.

### Detection of ROS level

DCFH-DA dye (Sigma) was employed to detect the intracellular ROS level. Cells were counted and planted into the 6-well plate at the density of 10^6^/well. After standing, cells were washed with PBS, and later medium containing 5 μM Oxaliplatin alone or various pathway regulators (Oltipraz 40 μM, ETC-1002 30 μM, liproxstatin-1 1 μM, and RSL3 0.5 μM) was added to culture for additional 6 h. After trypsin digestion, all cells in the wells were collected. Later, cells were centrifuged and washed repeatedly, followed by resuspension using the 4° C D-Hank’s solution until the concentration was 1×10^6^/ml. 100 μl cell suspension was collected and mixed with DCFH-DA to incubate for 20 min at 37° C in dark. Then, the non-specific dye was washed, and cells were analyzed and counted using the FACS Calibur flow cytometer.

### Lipid peroxidation detection

The intracellular lipid peroxidation level was evaluated using BODIPY-C11 (Invitrogen). Cells were incubated with 2.5 μM BODIPY-C11 fluorochrome for 10 min after treated with the above-mentioned drugs. Then, cells were washed with PBS for twice, followed by trypsin digestion and resuspension. Later, the intracellular fluorescence intensity was detected through flow cytometer (BD Biosciences, San Jose, CA, USA).

### GPX4 activity

Cells were collected in lysis buffer (100 mM Tris pH 7.6, 5 mM EDTA, 1 mM NaN3 and 0.1% peroxide-free Triton-X100). Lysates were complemented with 0.6 U/mL glutathione reductase, 0.2 mM nicotinamide adenine dinucleotide phosphate hydrogen, 3 mM reduced glutathione and 200 μM of the substrate cumene hydroperoxide. NADPH turnover was measured on an BioTek Synergy reader at 340 nm over 10 min at 37° C. Enzymatic activity was calculated after subtracting absorbance decay obtained from buffer without cell lysates by using NADPH extinction and by normalizing to total protein content [40].

### Real-time fluorescence quantitative PCR

The total RNA was isolated from the cultured cells or xenograft tissues directly using the Quick-RNA Miniprep Kit (Zymo, R1054) kit, which was later reversely transcribed using the HiScript IIQRT SuperMix for qPCR (+gDNA wiper) kit (Vazyme, China) in accordance with the manufacturer instruction for condition setting and step-by-step operation. The SYBR Green Master kit (Bio-Rad, USA) was used to process the samples for Q-PCR, and the Bio-Rad CFX384-Touch System (Bio-Rad) was used for detection. The gene expression level was calculated and presented according to the comparative CT method, as shown below: ∆∆CT=∆CT_sample_-∆CT_control_, fold change=2^-∆∆CT^. GAPDH expression was used as the internal reference.

### Western blotting

The intracellular protein was extracted using the lysis buffer, the concentration of total protein or protein after co-immunoprecipitation was quantified using the BSA method, and then loading buffer was added and boiled for degeneration. The loading amount of each sample was maintained at 50 μg. Later, the sample was subjected to sodium dodecyl sulfate-polyacrylamide gel electrophoresis (SDS-PAGE) for isolation, and then the protein was transferred onto the polyethylene (Bio-Rad, USA) membranes through the wet-transfer system. Then, the membranes were blocked with 5% skim milk prepared with TBST buffer to avoid the non-specific binding background, and incubated with primary antibody at 4° C overnight. On the following day, the membranes were washed before secondary antibody incubation at room temperature, and the protein bands were detected using the ChemiDoc™XRS+ system (Bio-Rad, USA). The Image J software was utilized to analyze the sample bands and calculated the results.

### Short hairpin (sh)RNA knockdown

Predesigned KIF20A-knockdown shRNA constructs were purchased from Sigma-Aldrich (Merck KGaA; cat. no. SHCLND-NM_005733_TRCN0000116522). Vehicle control construct was also provided by Sigma-Aldrich (Merck KgaA, cat. no. SHC016). The sequences for the human KIF20A-shRNA are 5’- CCGGCCTGAAG AAATAGGTCTCTTTCTCGAGAAAGAGACCTATTTCTTCAGGTTTTTG-3’. The plasmid (100ng/well) was transfected using Lipofectamine 2000 (Life Technologies) according to manufacturer’s instructions. The protein expression of KIF20A was detected by western blot assay to demonstrate the knockdown was successful.

### Statistical analysis

Data were expressed by the mean±SD of three independent experiments. The GraphPad Prism 7.0 software was utilized for statistical analysis. The significance of inter-group difference was determined through t-test and one-way analysis of variance (ANOVA). A difference of P<0.05 was deemed as statistically significant.

### Availability of data and material

We declared that materials described in the manuscript, including all relevant data, will be freely available to any scientist wishing to use them for non-commercial purposes, without breaching participant confidentiality.

## Supplementary Material

Supplementary Figures
