# Correction for: Suppressing the KIF20A/NUAK1/Nrf2/GPX4 signaling pathway induces ferroptosis and enhances the sensitivity of colorectal cancer to oxaliplatin

**DOI:** 10.18632/aging.203382

**Published:** 2021-07-26

**Authors:** Changshun Yang, Yu Zhang, Shengtao Lin, Yi Liu, Weihua Li

**Affiliations:** 1 Department of Surgical Oncology, Fujian Provincial Hospital, Fuzhou, 350001, China; 2 Department of Pathology, The First Affiliated Hospital of Fujian Medical University, Fuzhou, 350001, China; 3 Department of Endoscopy, National Cancer Center/Cancer Hospital, Chinese Academy of Medical Science and Peking Union Medical College, Beijing, 100000, China

**Keywords:** correction

Original article: Aging. 2021; 13:13515–13534.  . https://doi.org/10.18632/aging.202774

**This article has been corrected:** The authors requested to update Funding information. The authors declare that these corrections do not change the results or conclusions of this work.

