# Supplementary material for: Suppressing the KIF20A/NUAK1/Nrf2/GPX4 signaling pathway induces ferroptosis and enhances the sensitivity of colorectal cancer to oxaliplatin
Source: Aging (Albany NY). 2021 Mar 26;13(10):13515–34. doi: 10.18632/aging.202774 (PMC8202845; doi:10.18632/aging.202774)
Supplement: Supplementary Figures [file aging-13-202774-s001.pdf]

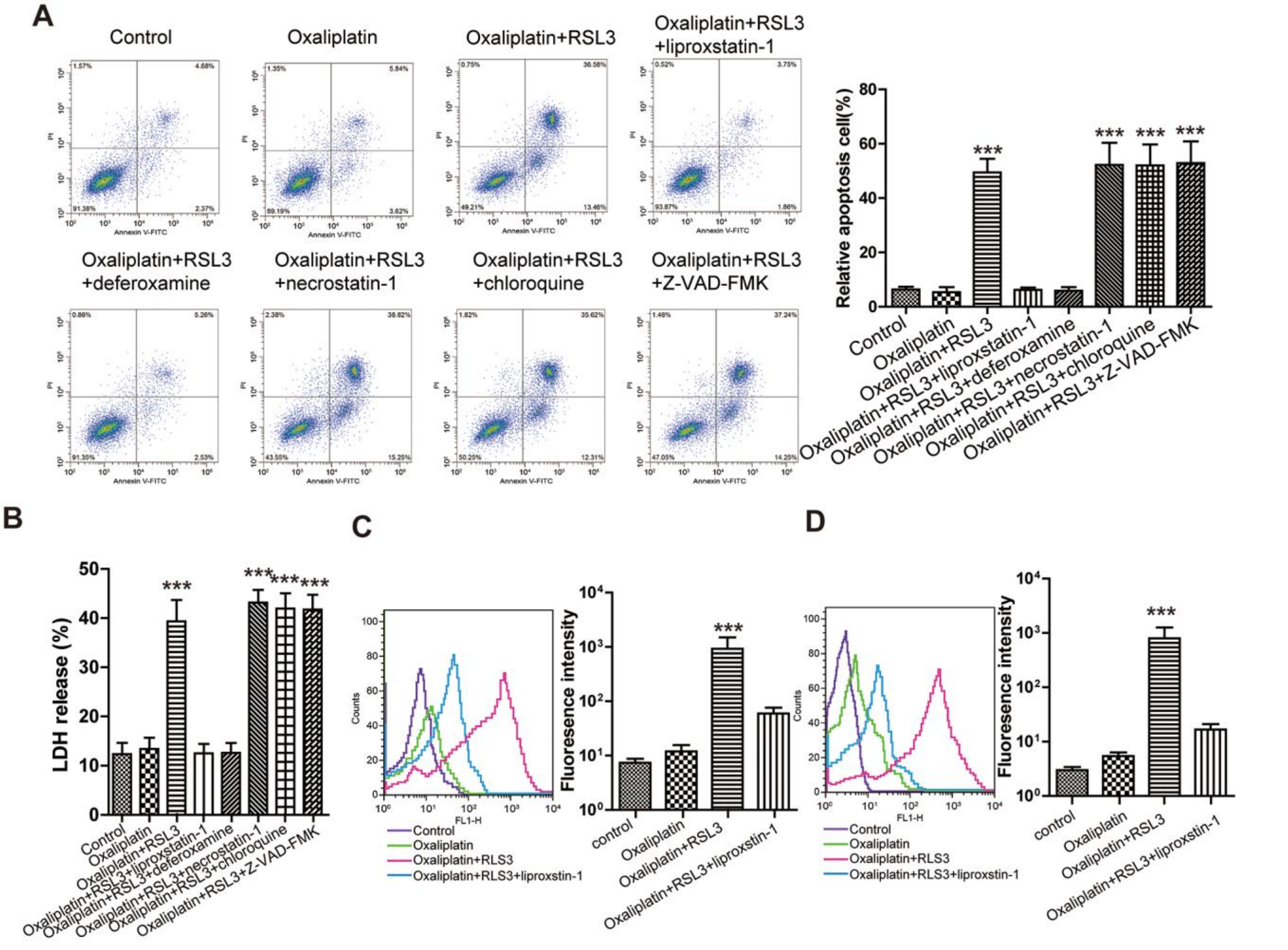

Supplementary Figure 1. Inducing ferroptosis enhanced the sensitivity of CRC to Oxaliplatin. (**A**) Cell death was assessed by flow cytometry (annexin V-FITC/PI staining) to observe whether RSL3 with or without the indicated inhibitors would affect the lethal effect of oxaliplatin on H716 cells *in vitro*. Left, representative results of annexin V-FITC/PI staining. Right, quantitative analysis. The data are presented as the mean ± SD, ***p < 0.001 (versus Oxaliplatin). (**B**) Cell death was assessed by LDH release assay to observe whether RSL3 with or without the indicated inhibitors would affect the lethal effect of oxaliplatin on H716 cells *in vitro*. The data are presented as the mean ± SD, ***p < 0.001 (versus Oxaliplatin). (**C**, **D**) The cellular level of ROS (**C**) and lipid peroxidation (**D**) was assessed by flow cytometry to observe whether RSL3 with or without liproxstatin-1 would affect the oxidative damage induction of oxaliplatin on H716 cells. The data are presented as the mean ± SD, ***p < 0.001 (versus Oxaliplatin).

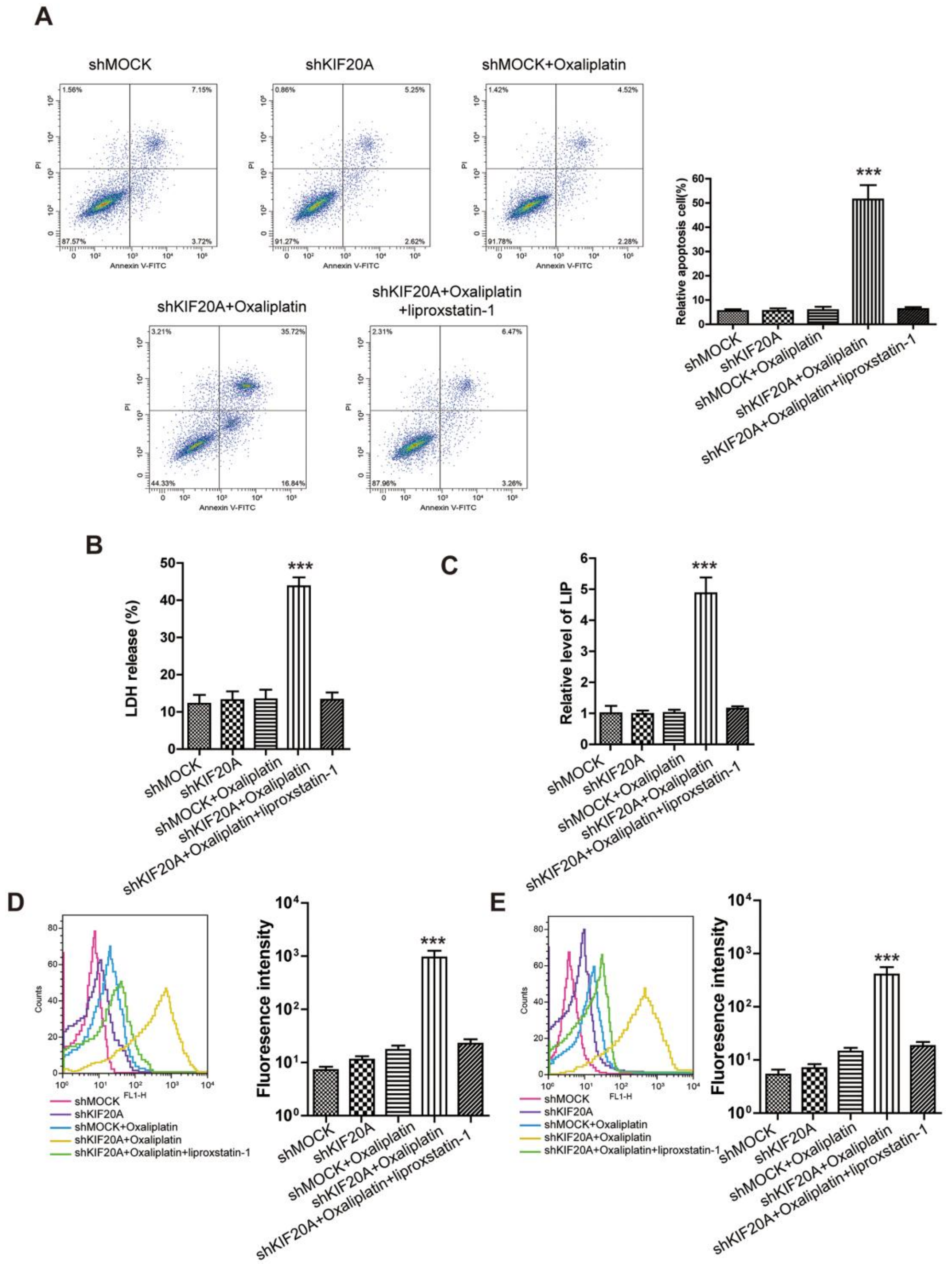

Supplementary Figure 2. High KIF20A expression in resistant CRC cell line suppressed the intracellular ferroptosis process. (**A**) Cell (H716) death was assessed by flow cytometry (annexin V-FITC/PI staining) to observe whether KIF20A silencing with or without liproxstatin-1 would affect the lethal effect of oxaliplatin on H716 cells *in vitro*. Left, representative results of annexin V-FITC/PI staining. Right, quantitative analysis. The data are presented as the mean ± SD, ***p < 0.001 (versus shMOCK+Oxaliplatin). (**B**) Cell (H716) death was assessed by LDH release assay to observe whether KIF20A silencing with or without liproxstatin-1 would affect the lethal effect of oxaliplatin on colorectal cancer *in vitro*. The data are presented as the mean ± SD, ***p < 0.001 (versus shMOCK+Oxaliplatin). (**C**) The cellular LIP was analyzed with a flow cytomete to observe whether KIF20A silencing with or without liproxstatin-1 would affect the LIP induction of oxaliplatin on H716 cells. The data are presented as the mean ± SD, ***p < 0.001 (versus shMOCK+Oxaliplatin). (**D**, **E**) The cellular level of ROS (**D**) and lipid peroxidation (**E**) was assessed by flow cytometry to observe whether KIF20A silencing with or without liproxstatin-1 would affect the oxidative damage induction of oxaliplatin on H716 cells. The data are presented as the mean ± SD, ***p < 0.001 (versus shMOCK+Oxaliplatin).

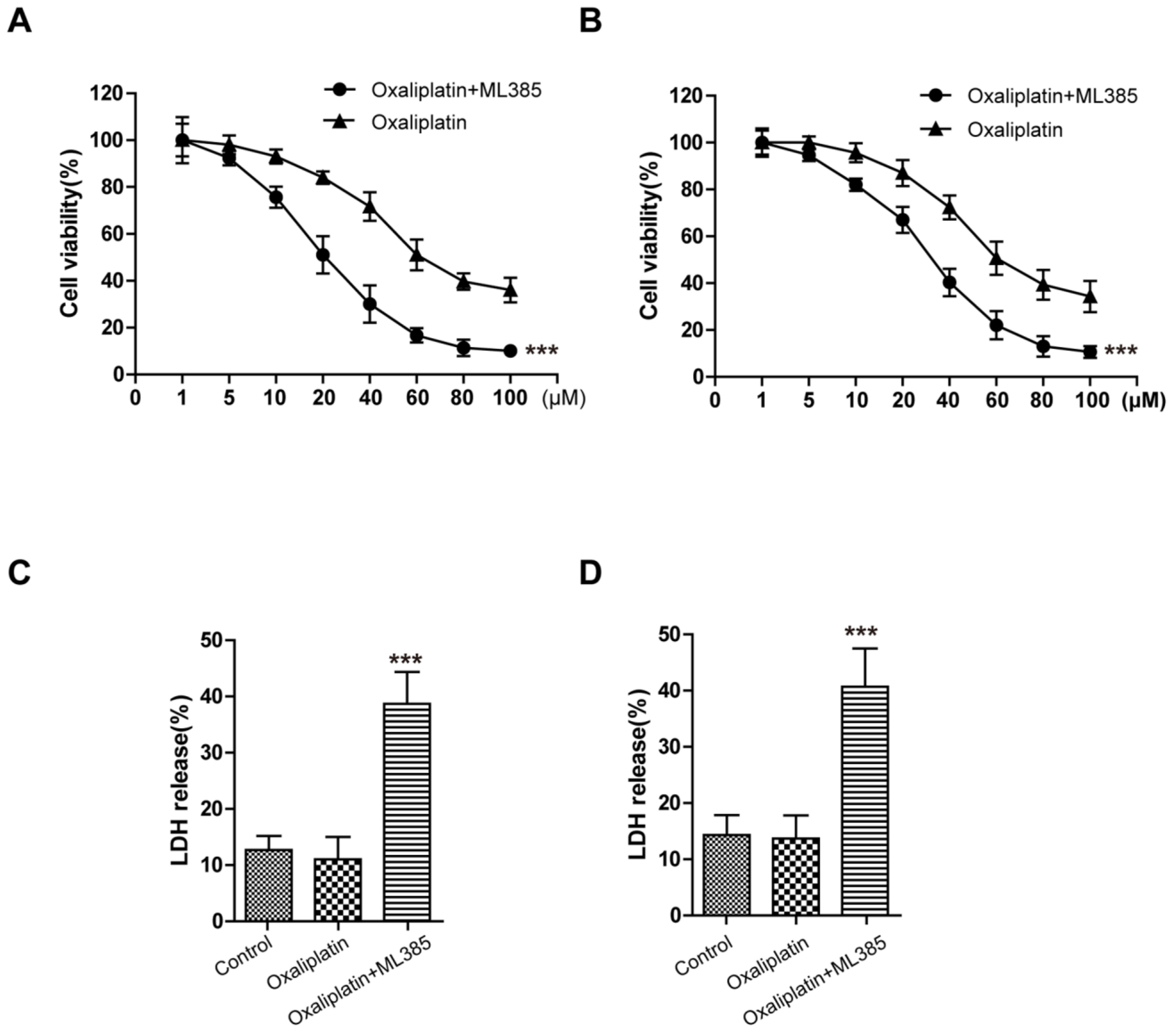

Supplementary Figure 3. The sensitivities of HCT116-Or and H716 to Oxaliplatin could be induced by the application of ML385. (**A**, **B**) The cell (HCT116-Or (**A**) and H716 (**B**)) viability was measured to observe whether ML385 would affect the suppression of oxaliplatin on resistant cancer cells *in vitro*. The data are presented as the mean ± SD, ***p < 0.001 (versus Oxaliplatin). (**C**, **D**) Cell death was assessed by LDH release assay to observe whether ML385 would affect the lethal effect of oxaliplatin on resistant cancer cells (HCT116-Or (**C**) and H716 (**D**)) *in vitro*. The data are presented as the mean ± SD, ***p < 0.001 (versus Oxaliplatin).
